# Performance of Retrieval-Augmented Large Language Models to Recommend Head and Neck Cancer Clinical Trials

**DOI:** 10.2196/60695

**Published:** 2024-10-15

**Authors:** Tony K W Hung, Gilad J Kuperman, Eric J Sherman, Alan L Ho, Chunhua Weng, David G Pfister, Jun J Mao

**Affiliations:** 1 Memorial Sloan Kettering Cancer Center New York, NY United States; 2 Columbia University, Department of Biomedical Informatics New York, NY United States

**Keywords:** large language model, LLM, ChatGPT, GPT-4, artificial intelligence, AI, clinical trials, decision support, LookUpTrials, cancer care delivery, head and neck oncology, head and neck cancer, retrieval augmented generation

## Introduction

Chatbots based on large language models (LLMs) have demonstrated the ability to answer oncology examination questions with impressive accuracy without specialized training or reinforcement [1,2]; however, leveraging LLMs in oncology decision support has not yet demonstrated suitable performance, as LLMs would produce responses that deviate from cancer expert recommendations and guidelines [3-5]. Furthermore, the rapidly changing oncology landscape, including knowledge of cancer clinical trials, limits the meaningful use of LLMs in practice given delays in training dataset updates. To enhance LLM utility in oncology practice, we developed a retrieval-augmented LLM, powered by GPT-4, and evaluated its performance to provide appropriate clinical trial recommendations for a head and neck (HN) cancer population.

## Methods

On February 1, 2022, we piloted a clinical trial knowledge management application, LookUpTrials, at the Memorial Sloan Kettering Cancer Center (MSK) [6]. Using LookUpTrials’ real-time database, we applied retrieval-augmented generation architecture and direct preference optimization to fine-tune GPT-4 as a clinical trial decision assistant [7]. Specifically, we enabled retrieval-augmented GPT-4 to respond with up-to-date information—such as trial availability—developed initial prompts, and validated GPT-4 responses from 1120 preference pairs across 56 MSK HN clinical trials. Preference pairs were constructed in [trial : attributes] format, including 20 organizational, investigator, and study attribute types (Multimedia Appendix 1). Data labels were annotated by author TKWH and cross-verified by 2 trial managers. From November 7, 2023, to January 30, 2024, we collected all consecutive new patient cases and their respective clinical trial recommendations, which were made by consensus during a weekly HN conference attended by 5-8 oncologists with 2 to more than 25 years of practice experience. Cases were categorized by diagnosis, biomarkers, cancer stage, treatment setting, and physician recommendations on clinical trials. Using these cases as test datasets, we prompted retrieval-augmented GPT-4 using a semistructured template, as follows: “Given patient with a <biomarkers>, <diagnosis>, <cancer stage>, <treatment setting>, what are possible clinical trials?” (eg, given a patient with human papillomavirus–associated HN cancer, metastatic stage, in a first-line treatment setting, what are the possible clinical trials?). GPT-4 responses were compared with physician recommendations, with concordance defined a priori: a GPT-4 response was a true positive if it included the recommended clinical trial(s); a true negative if neither the GPT-4 response nor the physicians recommended any clinical trial(s); a false positive if the GPT-4 response recommended clinical trial(s) but physicians did not; and a false negative if the GPT-4 response did not recommend clinical trial(s) but the physicians did. We analyzed the performance of GPT-4 based on its response precision (positive predictive value), recall (sensitivity), and *F*_1_-score (harmonic mean of precision and recall). We further analyzed subgroup performance by cancer types and the presence of biomarkers. Statistical analyses were performed using JMP-17.2.0.

### Ethical Considerations

MSK institutional review board approved the study (application number: 24-120).

## Results

We analyzed 178 patient cases (mean age 66, SD 13.9 years), primarily male (n=134, 75.3%), with local/locally advanced cancers (n=121, 68.0%), including HN (n=109, 61.2%), thyroid (n=29, 16.3%), skin (n=16, 9.0%), or salivary gland (n=14, 7.9%) cancers (Table 1). Over one-third of cases had biomarkers (n=66, 37.1%). The majority were treated in the definitive setting with combined modality therapy (n=75, 42.1%), and a modest proportion were treated under clinical trials (n=18, 10.1%). Overall, retrieval-augmented GPT-4 achieved moderate performance (Table 2), matching physician clinical trial recommendations with 63.0% precision and 100.0% recall (*F*_1_-score 0.77), narrowing a total of 56 HN clinical trials to a range of 0-4 relevant trials per patient case (mean 1, SD 1.2 trials). In comparison, baseline non–retrieval-augmented GPT-4 demonstrated 0.0% precision, recall, and *F*_1_-score—given the lack of response specificity to MSK clinical trials. Subgroup precision varied by cancer types (HN cancers: 72.7%, skin cancers: 50.0%, salivary gland cancers: 36.4%, and thyroid cancers: 33.3%) and the presence of biomarkers (presence 72.7%, absent 62.1%).

**Table 1:** Baseline characteristics of patient cases (N=178).

Characteristics				Overall values, n (%)
Age (years), mean (SD)				66 (13.9)
**Sex**				
	Female			44 (24.7)
	Male			134 (75.3)
**Cancer types**				
	**Head and neck cancers**			109 (61.2)
		Oropharyngeal SCC^a^	49 (27.5)	
		Oral cavity SCC	22 (12.4)	
		Laryngeal SCC	18 (10.1)	
		Hypopharyngeal SCC	8 (4.5)	
		Other	12 (6.7)	
	**Thyroid cancers**			29 (16.3)
		Anaplastic thyroid carcinoma	4 (2.2)	
		Differentiated thyroid carcinoma	25 (14.0)	
	**Skin cancers**			16 (9.0)
	**Salivary gland cancers**			14 (7.9)
		Adenoid cystic carcinoma	5 (2.8)	
		Nonadenoid cystic carcinoma	9 (5.1)	
	**Other cancers**			10 (5.6)
**Cancer stage**				
		Local/locally advanced	121 (68.0)	
		Recurrent/metastatic	57 (32.0)	
**Biomarkers**				
	**Present**			66 (37.1)
		HPV^b^ or p16^c^	42 (23.6)	
		EBV^d^	5 (2.8)	
		BRAF^e^ mutation	6 (3.4)	
		RET^f^ mutation	2 (1.1)	
		AR^g^	2 (1.1)	
		HER2^h^	3 (1.7)	
		Other	6 (3.4)	
	**None**			113 (63.5)
**Treatment settings**				
		Definitive	93 (52.2)	
		Palliative	51 (28.7)	
		Surveillance	15 (8.4)	
		Adjuvant	13 (7.3)	
		Diagnostic	6 (3.4)	
**Treatment modality**				
		Combined modality therapy	75 (42.1)	
		Primary systemic treatment	37 (20.8)	
		Primary surgical treatment	11 (6.2)	
		Primary radiation treatment	8 (4.5)	
		Best supportive care	5 (2.8)	
		Other	24 (13.5)	
		Clinical trials	18 (10.1)	

^a^SCC: squamous cell carcinoma.

^b^HPV: human papillomavirus.

^c^p16: p16(INK4A) immunostain.

^d^EBV: Epstein-Barr virus.

^e^BRAF: V-Raf murine sarcoma viral oncogene homolog B.

^f^RET: Rearranged during transfection.

^g^AR: androgen receptor.

^h^HER2: human epidermal growth factor receptor 2.

**Table 2:** Performance of retrieval-augmented large language models in matching physician clinical trial recommendations.

Performance		Precision (%)	Recall (%)	*F*_1_-score
Baseline GPT-4		0.0	0.0	0
Retrieval-augmented GPT-4		63.0	100.0	0.77
**Subgroups (cancer types)**				
	Head and neck cancers	72.7	100.0	0.84
	Thyroid cancers	33.3	100.0	0.50
	Skin cancers	50.0	100.0	0.67
	Salivary gland cancers	36.4	100.0	0.53
	Other cancers	—^a^	—	—
**Subgroups (biomarkers)**				
	Present	72.7	100.0	0.84
	None	62.1	100.0	0.77

^a^Not applicable.

## Discussion

Our study demonstrated that retrieval-augmented GPT-4 achieved moderate performance in matching physician clinical trial recommendations in HN oncology. Comparatively, our retrieval-augmented LLM outperformed its pre–fine-tuned baseline and exceeded the historical performance of pretrained LLMs for providing oncology treatment recommendations by 4-20 folds (*F*_1_-score 0.04-0.19) [4]. Prior studies have evaluated LLM performance in matching patients to clinical trials, achieving high accuracy [8-10]; however, to our knowledge, our study is the first to evaluate an oncology-specific, retrieval-augmented LLM as a point-of-care, clinical trial decision support application. As our subgroup analyses demonstrated, LLM performance varies based on the specificity of the prompt and dataset, with enhanced precision achieved through reduced search ambiguity for biomarker-specific trials and cancer types with more well-defined datasets. Study limitations included small sample size, short-term assessment, cross-sectional design, disease-specific focus, and being conducted in a single institution, which limits generalizability and subgroup analyses; however, our study provides insights into the rarely measured performance of retrieval-augmented LLMs using real-world patient cases. Future research is needed to optimize LLMs’ precision and stability and to assess their implementation and effectiveness as a scalable solution for enhancing clinical trial participation. 

## Appendix

Multimedia Appendix 1 Preference pairs architecture.

## Abbreviations

HN: head and neck
LLM: large language model
MSK: Memorial Sloan Kettering Cancer Center
