# Supplementary material for: Performance of Retrieval-Augmented Large Language Models to Recommend Head and Neck Cancer Clinical Trials
Source: J Med Internet Res. 2024 Oct 15;26:e60695. doi: 10.2196/60695 (PMC11522650; doi:10.2196/60695)
Supplement: Multimedia Appendix 1 [file jmir_v26i1e60695_app1.docx]

| **Table S1.** [Trial : Attributes] Preference Pairs Architecture | | | |
| --- | --- | --- | --- |
| **Attributes** | **Trial #1** | **Format** | **Example** |
| Organization ID | MSK | [MSK, ...] | MSK |
| Departmental ID | HN | [HN, ...] | HN |
| Trial Number | Trial #1 | [#, ...] | 19-00891 |
| PI Name(s) | PI Names | [[Names], ...] | Jane Bruin |
| PI Email(s) | PI Emails | [[Emails], ...] | jbruin@email.org |
| CRA Name(s) | CRA Names | [[Names], ...] | Mike Bear |
| CRA Email(s) | CRA Names | [[Emails], ...] | [mbear@email.org](mailto:mbear@email.org) |
| Trial Title | Title #1 | [Title, ...] | Phase 2 study of drug A |
| Condition(s) or Diagnoses | Diagnoses ... | [[Diagnoses], ...] | Oropharyngeal cancer |
| Stage(s) | Stages... | [[Stages], ...] | Metastatic |
| Treatment Setting(s) | Treatment Settings... | [[Treatment Settings], ...] | First-line treatment |
| Biomarker(s) | Biomarkers... | [[Biomarkers], ...] | HPV |
| Special Consideration(s) | Special Considerations... | [[Special Considerations] ...] | Elderly |
| Symptom(s) | Symptoms | [[Symptoms], ...] | Fatigue |
| Keywords (5) | Keywords | [[Keywords], ...] | HN Cancer |
| NCT Number | NCT #1 | [NCT#, ...] | NCT5729300019 |
| MSK: Memorial Sloan Kettering Cancer Center  HN: Head & Neck Oncology Department  PI: Principal Investigator  CRA: Clinical Research Assistant  HPV: Human Papilloma Virus  NCT: National Clinical Trial | | | |
